# Simulating Fiction: Individual Differences in Literature Comprehension Revealed with fMRI

**DOI:** 10.1371/journal.pone.0116492

**Published:** 2015-02-11

**Authors:** Annabel D. Nijhof, Roel M. Willems

**Affiliations:** 1 Donders Institute for Brain, Cognition and Behavior, Radboud University Nijmegen, Nijmegen, The Netherlands; 2 Max Planck Institute for Psycholinguistics, Nijmegen, The Netherlands; Max Planck Institute for Human Cognitive and Brain Sciences, GERMANY

## Abstract

When we read literary fiction, we are transported to fictional places, and we feel and think along with the characters. Despite the importance of narrative in adult life and during development, the neurocognitive mechanisms underlying fiction comprehension are unclear. We used functional magnetic resonance imaging (fMRI) to investigate how individuals differently employ neural networks important for understanding others’ beliefs and intentions (mentalizing), and for sensori-motor simulation while listening to excerpts from literary novels. Localizer tasks were used to localize both the cortical motor network and the mentalizing network in participants after they listened to excerpts from literary novels. Results show that participants who had high activation in anterior medial prefrontal cortex (aMPFC; part of the mentalizing network) when listening to mentalizing content of literary fiction, had lower motor cortex activity when they listened to action-related content of the story, and vice versa. This qualifies how people differ in their engagement with fiction: some people are mostly drawn into a story by mentalizing about the thoughts and beliefs of others, whereas others engage in literature by simulating more concrete events such as actions. This study provides on-line neural evidence for the existence of qualitatively different styles of moving into literary worlds, and adds to a growing body of literature showing the potential to study narrative comprehension with neuroimaging methods.

## Introduction

Narratives play an important role in human life, and it is more and more acknowledged that fiction is a powerful player in human development as well as in adulthood (e.g. [1,2,3]). Despite its importance, it is largely unknown what the brain networks are that support our unique ability to move into a fiction world. While it is uncontroversial that people *are* moved into fiction worlds [4,5], it is unclear *how* readers do this. People differ greatly in how they engage in fiction (e.g. [6,7–11]), but the neurocognitive mechanisms behind narrative engagement remain unclear (see [12] for related work on theatre). Here we use neuroimaging to investigate individual differences during the comprehension of literary fiction stories.

One way in which participants engage with stories, is via simulation of the story’s content. Recent philosophical and neuroscientific evidence shows that it is important to distinguish at least two neurocognitively distinct components of simulation when considering the understanding of narratives (e.g. [13]). First, *sensori-motor simulation* is evidenced by activation of motor and visual cortices when people comprehend language related to actions and scenery [14–17]. The second component relates to our ability to understand thoughts, intentions and beliefs of others, sometimes called *mentalizing* [18]. The distinction between these two components important for fiction understanding is theoretically motivated (e.g. [13]), and supported by neural findings (e.g. [19,20]).

In this study participants listened to excerpts (4 to 8 minutes long) from literary novels, while neural activity was measured across the whole brain by means of fMRI. We chose to use listening rather than word-by-word reading, because relatively long fragments are used and therefore listening is expected to be the most convenient option for participants in the scanner. Supposedly, this would not result in crucial differences in terms of the mental simulation they employ. Previous studies did find differences in brain activity between listening and reading (with more individual differences in activity for reading), but also several core regions shared between modalities [21]. More importantly for the purposes of the present study, it has been shown that regions involved in mentalizing [22] and action understanding [23] are activated independent of presentation modality. It is an open question whether mentalizing differs during reading or listening to narratives, but based on the previous literature we expect the two modalities to engage overlapping neural correlates.

Stories were tagged for motor (‘action’) and mentalizing content, and memory for the stories was debriefed afterwards. Brain regions known to be involved in the two kinds of simulation were localized with standardized localizer tasks. Target regions were left and right motor regions for sensori-motor simulation [24], and anterior medial prefrontal cortex (amPFC), right temporoparietal junction (rTPJ) and precuneus for mentalizing [19,25] (see methods section). Importantly, measurement of brain activity during story comprehension was done on-line, and without additional tasks for the listener.

Some recent neuroimaging studies relate to the issue of mentalizing and sensori-motor simulation during the comprehension of narratives. For instance, Wallentin and colleagues showed that part of the visual cortex which is sensitive to perceiving visual motion, is also activated when participants heard pieces describing movement in a retelling of ‘The Ugly Duckling’ [26]. Similarly, Speer and colleagues showed that parts of short children’s stories containing action descriptions activated the motor cortex [15]. In an interesting recent approach, Altmann and colleagues presented short stories (around 40 words per story) to participants. Stories were either labeled as fact (describing an event that actually took place) or as fiction. Most interesting for the current approach was that stories that were labeled as fiction led to stronger activation in medial prefrontal cortex (among other regions), whereas labeling stories as describing actual facts led to higher activation levels in the premotor cortex (again, amongst other regions) ([27]; see also [14,28]).

In this study we follow up on this previous work by using more extended (i.e. longer) excerpts from literary fiction, written for adults, in order to give participants an experience of engaging with fiction that is relatively close to their real-world experience. We have a special focus on individual differences in simulation and mentalizing during narrative comprehension. Previous work has found that participants differ in how much they engage parts of the mentalizing system during the reading of texts labeled as fiction [27], as well as that participants differ in how much they engage in sensori-motor simulation during language comprehension [29,30]. Here we combine these two to see how participants differ in their engagement of these two important subprocesses of narrative comprehension while listening to natural, unmodified literary fiction.

## Materials and Methods

### Participants

Eighteen healthy, naïve native speakers of Dutch without psychiatric or neurological problems, and with normal or corrected-to-normal vision and no hearing problems took part in the experiment. Four participants were male, fourteen female. The average age was 22.2 years (range 18–27). Data for the ‘Theory-of-Mind localizer’ of one participant showed artifacts (Nyquist ghosting) and were therefore removed. Written informed consent was obtained prior to the study, and ethical approval was obtained from the local ethics committee (CMO Committee on Research Involving Human Subjects, Arnhem-Nijmegen, The Netherlands, protocol number 2001/095), in line with the Declaration of Helsinki. Participants were paid either in money or in course credit at the end of the study.

### Story stimuli

Sound recordings of three literary stories were selected from the Corpus of Spoken Dutch (‘Corpus Gesproken Nederlands’, [31]). Recordings were spoken at a normal rate, in a quiet room by female speakers (one speaker per story). The fragments were taken from literary novels, and all contained descriptions of actions, characters, scenery, and plot and character development. Duration of the fragments was 3:49, 7:50, or 7:48 minutes, and the number of words was 622, 1291, and 1131 words per story. In order to create an experimental baseline condition, reversed speech versions of the story fragments were created (using Audacity 2.03, http://audacity.sourceforge.net).

The story fragments were annotated for Action and for Mentalizing content over the course of the story. Quantification of the content at specific time points in the story was done with PRAAT [32], and consisted of assigning sentence parts which contained Action descriptions or Mentalizing descriptions to either category. A sentence part was coded as Action if it contained action of a person or an object. A sentence part was coded as Mentalizing if a character’s mental states (emotions, desires, intentions and/or beliefs) were described, as well as when a character was described in terms of his or her personality (Table 1). Sentence parts were predefined in the corpus, and never contained more than one main verb. Taken together, the Mentalizing sentence parts made up 22.9% of the total story durations, and the Action sentence parts 25.6%. A sentence part could contain more than one kind of description: the two descriptors had 11.1% overlap. Mean duration of the Mentalizing events (sentence parts) was 1.58 seconds (s.d. 0.76 sec.), and 1.75 seconds for Action events (s.d. 0.70 sec.). In total there were 252 Mentalizing events, and 225 Action events.

**Table 1:** Example of stimulus scoring.

The animal was skinny, and its round eyes bulged out of its head, ***as if it still hadn’t recovered from the anxiety caused by its own sudden death***. He searched for a stick and pushed the rabbit over: as stiff as a board.

Translated excerpt from one of the stories, containing Action content (underlined), as well as Mentalizing content (in bold italics). All three stories were scored in a similar fashion, leading to regressors for the fMRI analysis for Action content, and for Mentalizing content. See the Stimuli section for more information.

### Procedure

**Main task**. The main task was always carried out first. Participants were auditorily presented with the three story fragments while they were lying in the MRI scanner. Intermixed with the stories, the three reversed speech versions of the stories were played. There was no additional task but to listen to the materials carefully and attentively. There was a short break after each fragment. The stories were presented in counterbalanced order across participants, with the reversed speech version always being played either before or after the specific story it was created from.

**Localizers for regions of interest**. After presentation of the stories, localizer scans were taken to define regions of interest (ROIs) that were hypothesized to be active during either motor simulation or during mentalizing. ROIs for motor simulation were defined by having participants carry out a localizer task for action execution. Simple hand action was required (opening and closing of the fingers of both hands)—a fast method that has proven to reliably elicit the motor cortex in studies of action language comprehension [17,33]. Each trial started with a fixation cross presented for 1 s, followed by one of the words HANDS or REST for 10 s in white text on a black screen. Participants were required to continuously open and close their hands (Hand blocks), or to not move (Rest blocks). There were six of these trials per condition. The trials were presented in a pseudorandom order so that a specific condition was not presented more than twice subsequently.

The task that was used to define mentalizing ROIs was based on an existing Theory-of-Mind localizer [25]. Here, the version designed by Dodell-Feder, Koster-Hale, Bedny and Saxe was used, in a Dutch translation [34,35]. The task consists of a ‘false belief story’ task that activates regions that are known to be specifically activated when thinking about other persons’ beliefs and intentions. Followed by an initial instruction screen that stayed up until button press, participants read 20 short stories, divided in two blocks of ten. Each trial started with a fixation cross that was presented for a time interval randomly jittered between 4 and 8 s, in steps of 250 ms (intertrial interval). Then a story of two or three sentences was presented in white text on a black screen for 10 s. Each story was followed by a single-sentence statement that could be true or false. This statement stayed on the screen for 5 s, and within this time participants had to make a left button press for true, right for false statements. Half of the stories belonged to a *false photograph* (non-Mentalizing), half to a *false belief* (Mentalizing) condition: regions were defined on the basis of this contrast. That is, they reflected where activity was greater for the false belief condition than for the false photograph condition. The stories were presented in pseudorandom order, so that the same condition was never presented more than two times in a row.

On the basis of the two localizer tasks, regions of interest were created. This was done for the action localizer, by taking significant clusters from the ‘hands > rest’ contrast, using a statistical threshold of p<0.05 Family Wise Error-corrected at the voxel level. For the Mentalizing localizer, the contrast ‘false belief > false photograph’ (story plus subsequent test statement together) was used. Results were corrected for multiple comparisons by combining a voxel-wise threshold of p<0.001 with a cluster extent threshold computed using the theory of Gaussian random fields, to arrive at a statistical threshold with a p<0.05 significance level [36]. The different procedure for statistical thresholding was motivated by the fact that action execution leads to very strong and easily detectable activations, warranting a more conservative thresholding procedure. The mean brain activity in each ROI (technically the mean beta weights per condition) during story comprehension was extracted per regressor (Mentalizing content, Action content) per participant, using the SPM toolbox MarsBaR [37].

**Post-hoc memory test**. Participants, once out of the scanner, got a surprise test to check their memory for each of the stories. Participants were not told about this memory test before the start of the experiment. There were five multiple-choice questions per story fragment, with three possible answers (A, B, C) each. The questions were asking for general content, varying in level of detail (Example: What could be seen at the horizon? A. wind mill, B. watchtower, C. radio mast). Memory scores were summed, providing an overall score of participants’ memory and on-line attention to the story.

### fMRI data acquisition and preprocessing

Images of blood-oxygen level dependent (BOLD) changes were acquired on a 3T Siemens Magnetom Trio scanner (Erlangen, Germany) with a 32-channel head coil. Pillows and tape were used to minimize participants’ movement, and earphones used for presenting the stories also minimized scanner noise. Functional images were acquired using a fast T2*-weighted 3D EPI sequence [38], with high temporal resolution (TR: 880 ms, TE: 28 ms, flip angle: 14 degrees, voxel size: 3.5 x 3.5 x 3.5 mm, 36 slices). High resolution (1 x 1 x 1.25 mm) structural (anatomical) images were acquired using an MP-RAGE T1 GRAPPA sequence.

Preprocessing was performed using the Matlab toolbox SPM8 (http://www.fil.ion.ucl.ac.uk/spm). After removing the first four volumes to control for T1 equilibration, images were motion corrected and registered to the first image. The mean of the motion-corrected images was then coregistered with the individual participants’ anatomical scan. The anatomical and functional scans were spatially normalized to the standard MNI template. Finally, all data were spatially smoothed using an isotropic 8 mm full width at half maximum (FWHM) Gaussian kernel.

### Stimulus presentation

Stimuli were presented with Presentation software (version 16.2, http://www.neurobs.com). Auditory stimuli were presented through MR-compatible earphones. Presentation of the story fragments was preceded by a volume test: a fragment from another story but with comparable voice and sound quality was presented while the scanner was collecting images. Volume was adjusted to the optimal level based on feedback from the participant. All visual stimuli were projected onto a screen using a projector outside the MR scanner room, which could be seen by participants through a mirror mounted over the head coil. Responses to the Mentalizing localizer task were recorded with two button boxes (left and right hand). The story parts of the experiment required no response (listening only).

### Data analysis

At the single-subject level, statistical analysis was performed using a general linear model, in which beta weights for each regressor of interest are estimated using multiple regression analysis [39]. In this model, the two regressors of interest (‘Mentalizing’ descriptions and ‘Action’ descriptions) were modeled as their true durations, convolved with a canonical hemodynamic response function [40]. The variance inflation factor (VIF) of the two regressors of interest was calculated, to ensure that unique variance could be attributed to each regressor. The VIF of ‘Mentalizing’ and ‘Action’ was 1.22, which is low, and well within the range for assessing multicollinearity, in which values bigger than 10 are problematic [41–43]. The motion estimates of the motion correction algorithm (linear, quadratic and first-derivative regressors for three translations and three rotations) were modeled as regressors of no interest to account for head motion.

To address the question about individual differences in kinds of simulation, activity for the Action regressor in action ROIs was correlated with activity for the Mentalizing regressor in mentalizing ROIs. The rationale behind this analysis is that if people differ in whether they engage more in one type of simulation compared to the other, we should find that there is a relationship between activity in the mentalizing network during Mentalizing descriptions, and activity in the neural action network during Action descriptions. All correlations were two-sided Pearson’s correlations, and to guard against false positives, all correlation results were corrected for the number of comparisons by changing the critical alpha value using Bonferroni correction.

As a control analysis, the same correlation analysis was repeated on the data acquired while participants listened to the reversed speech fragments, for which the Mentalizing and Action regressors are meaningless. Naturally, no significant correlations were expected between regions and different regressors in this analysis. A direct comparison between correlation values of the real and reversed speech sessions was done using Steiger’s test [44].

To see if the action ROIs and the mentalizing ROIs were each co-activated during story comprehension, Action regressor activity in each of the action ROIs was correlated with the other action ROIs, and Mentalizing regressor activity in each of the mentalizing ROIs was correlated with the other mentalizing ROIs. We expect the ROIs for each condition to co-activate, in line with previous studies (see e.g. [45] for action ROIs, and [46,47] for mentalizing ROIs).

Finally, as another control analysis, activity for the Action regressor in mentalizing ROIs was correlated with activity for the Mentalizing regressor in action ROIs. No relationship between those regressors and ROI activity was expected, and therefore also no significant correlations between the mentalizing and action ROIs.

Although our main hypothesis concerned the mentalizing and action neural networks, we additionally performed a whole-brain analysis to assess whether there were activations of interest outside of our target networks. Statistical group analysis was performed by directly contrasting one of the sentence part regressors with the other. Participants were treated as a random factor in this analysis (“random effects model”, [48]). Results were corrected for multiple comparisons by combining a voxel-wise threshold of p<0.001 with a cluster extent threshold computed using the theory of Gaussian random fields to arrive at a statistical threshold of p<0.05 [36].

## Results

### Behavioral

Participants answered on average 9.9 (s.d. 1.11) questions correct of the 15 questions asked in the post-hoc memory questionnaire (multiple choice, three alternatives, 5 questions per story). Participants performed well above chance (p<0.001 for all stories) on the memory test, and there were no differences between the three stories (F(2, 16) = 1.41, p = 0.27; mean story 1: 3.17 (s.d. 1.25), story 2: 3.67 (s.d. 1.09), story 3: 3.11 (s.d. 0.96)). This indicates that participants paid attention to the story content, and that this was equally the case for all three stories.

### Localizers

The results of the localizer tasks show that the localizers worked well: both revealed activations in sets of areas that were expected based on previous literature (see below).

**Fig 1:**
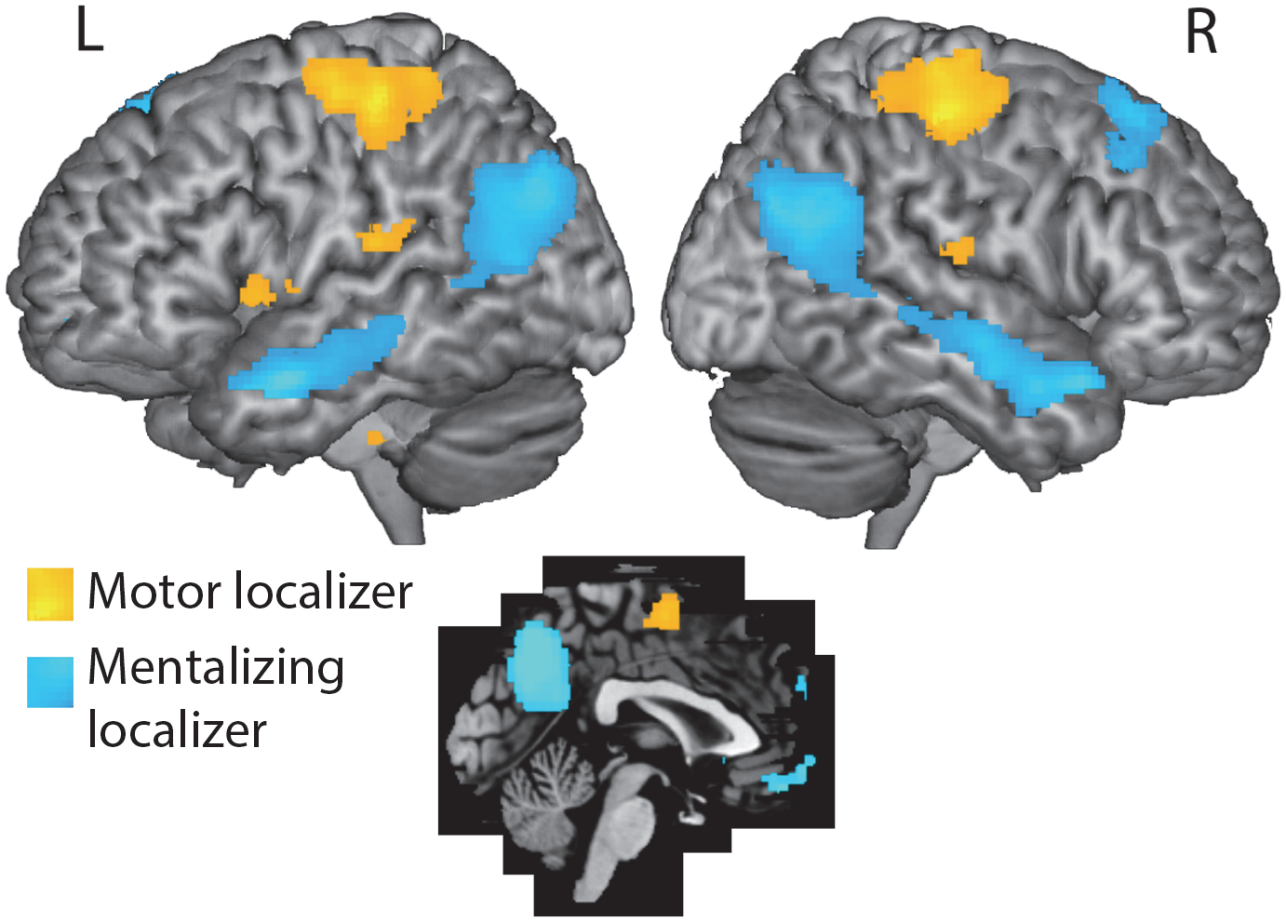
Results of the localizer scans. Whole-brain analysis results for the action localizer scan in yellow (hand action execution versus rest), and for the mentalizing localizer in blue (false belief stories versus false photograph stories [34]). The action localizer activated the cortical motor system robustly, and the mentalizing localizer led to activations in the previously defined mentalizing (or Theory-of-Mind) network. Areas from the localizers were used in the main analysis as regions of interest. Results are displayed at a statistical threshold level of p<0.05, corrected for multiple comparisons.

**Action localizer**. The action localizer activated the cortical motor system robustly. The ‘hands > rest’ contrast resulted in activations in the precentral and central motor regions bilaterally, as well as in the supplementary motor area (SMA), and in the cerebellum (Fig. 1). Since we had no a priori hypothesis about distinctions within the motor system during sensori-motor simulation, and to reduce the number of tests in the correlation analysis, we combined the cortical ROIs into two motor cortex ROIs, one for the left hemisphere, and one for the right hemisphere (MNI coordinates of centre voxel: left -34 -23 56, right 32 -20 58). This means the cerebellum was excluded from further analysis.

**Mentalizing localizer**. The regions of interest defined from the Mentalizing localizer’s ‘false belief > false photograph’ contrast were the medial prefrontal cortex, left and right temporo-parietal junction, left and right middle temporal gyrus, and the precuneus (Fig. 1). Given previous research, and in order to restrict the number of statistical tests, we selected, as mentioned above, the mentalizing ROIs from the results that are most commonly reported in previous literature, namely aMPFC (MNI coordinates -3 50 -10, rTPJ 51 -60 25, and precuneus -1 60 37 [19,25]).

### ROI analysis

**Fig 2:**
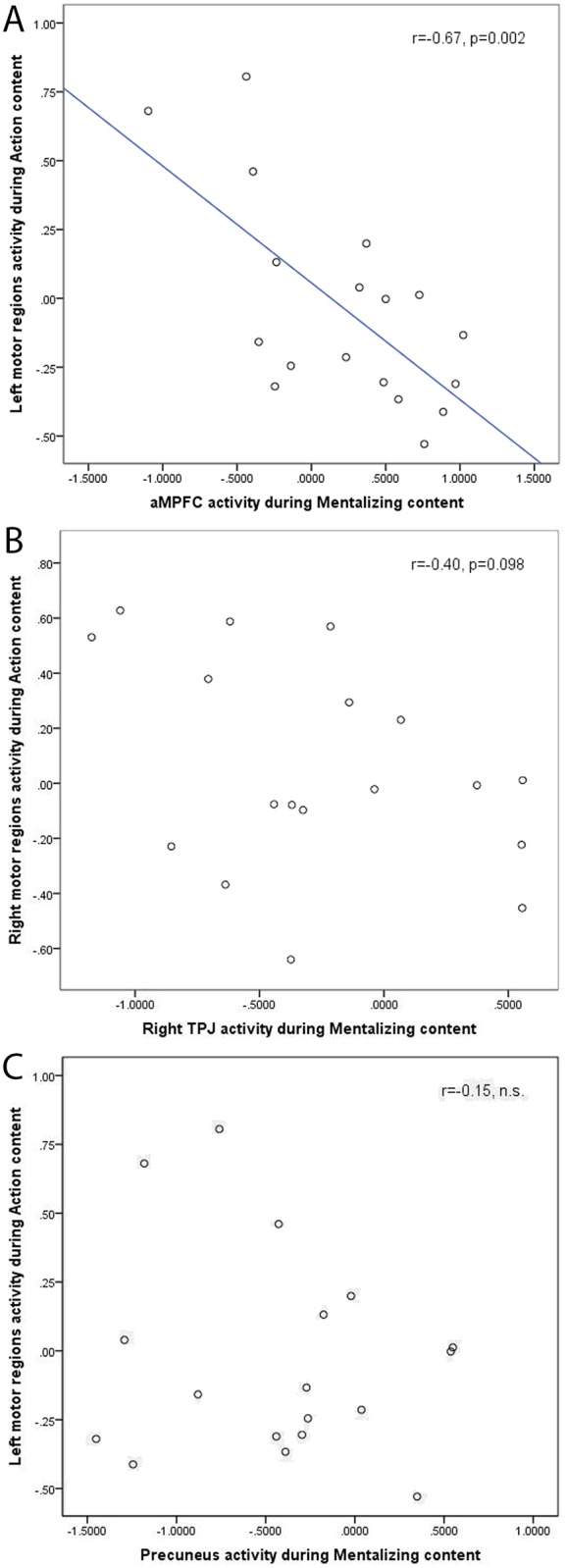
Results of the correlation analysis. Scatter plots of activation levels (beta weights) of Mentalizing regions (x-axes) while participants listened to mentalizing content, and activation in Motor regions while participants listened to Action content (y-axes). **A**) There is a negative correlation between Mentalizing regressor activity in the aMPFC mentalizing ROI (x-axis) and Action regressor activity in the left precentral action ROI (y-axis). This illustrates the individual differences in engaging with fiction, with a gradient going from those who engage exclusively in mentalizing, to participants that engage much more in motor simulation (Table 2). **B**) Relationship between Mentalizing activation in right TPJ and Action content activation in right motor cortex. There is a negative relationship, which is sizeable (r = -0.40), but does not reach statistical significance. **C**) No relationship was observed between Mentalizing activation in the precuneus and Action content in left Motor regions. Activity is expressed as the mean (over voxels in a ROI) of beta weights of a specific regressor in the regression model. The beta weights reflect the fit of BOLD activity with the modeled response to either Action or Mentalizing events. Every dot represents activation from one participant.

Mentalizing regressor activity in the mentalizing ROIs was correlated with Action regressor activity in the action ROIs. A significant negative correlation was found between the action ROIs activation for Action descriptions, and the aMPFC mentalizing ROI activity for Mentalizing descriptions (Fig. 2A; Table 2). This shows that participants who engaged the aMPFC when listening to mentalizing content, did engage the cortical motor system less when listening to Action descriptions, and vice versa. Importantly, the same correlations were not observed for the reversed speech data (all p>0.5; Table 3). A direct comparison using Steiger’s Z-test [44] of the correlation values showed that correlations between aMPFC and motor regions during the real speech fragments were more negative than during the reversed speech fragments (z = -1.74, p = 0.04). A similarly negative correlation was found between right TPJ activity during mentalizing content and right motor cortex during action content (Fig. 2B), and although this effect did not reach statistical significance, it is still sizeable (r = -0.40), and importantly in the same direction as the aMPFC-motor cortex correlation. No significant correlation was observed between Mentalizing activation in the precuneus, and Action-related activation of either of the Motor regions (Fig. 2C).

**Table 2:** Correlations between mentalizing and action regions.

Mentalizing region	Action region	r	p-value
aMPFC	Left motor regions	**-0.670**	**0.002**
	Right motor regions	**-0.640**	**0.004**
Right temporo-parietal junction	Left motor regions	-0.257	0.302
	Right motor regions	-0.402	0.098
Precuneus	Left motor regions	-0.148	0.559
	Right motor regions	-0.262	0.293

Correlations between Mentalizing regressor activity in mentalizing regions and Action regressor activity in action regions. The results indicate that activity in the aMPFC while participants listened to Mentalizing content, correlated negatively with activity in action regions while participants listened to Action-related content (see Fig. 2 for an illustration). The r and p-values in bold typeface indicate significant correlations at the Bonferroni-corrected critical alpha level, and hence indicate results which are corrected for multiple comparisons. The responses in parts of the cortical motor network were summed over motor regions, as described in the methods section.

**Table 3:** Correlations between mentalizing and action regions for the reversed speech control data.

Mentalizing region	Action region	r	p-value
aMPFC	Left motor regions	-0.150	0.540
	Right motor regions	-0.070	0.782
Right temporo-parietal junction	Left motor regions	-0.127	0.614
	Right motor regions	0.063	0.805
Precuneus	Left motor regions	-0.122	0.630
	Right motor regions	-0.029	0.910

**Fig 3:**
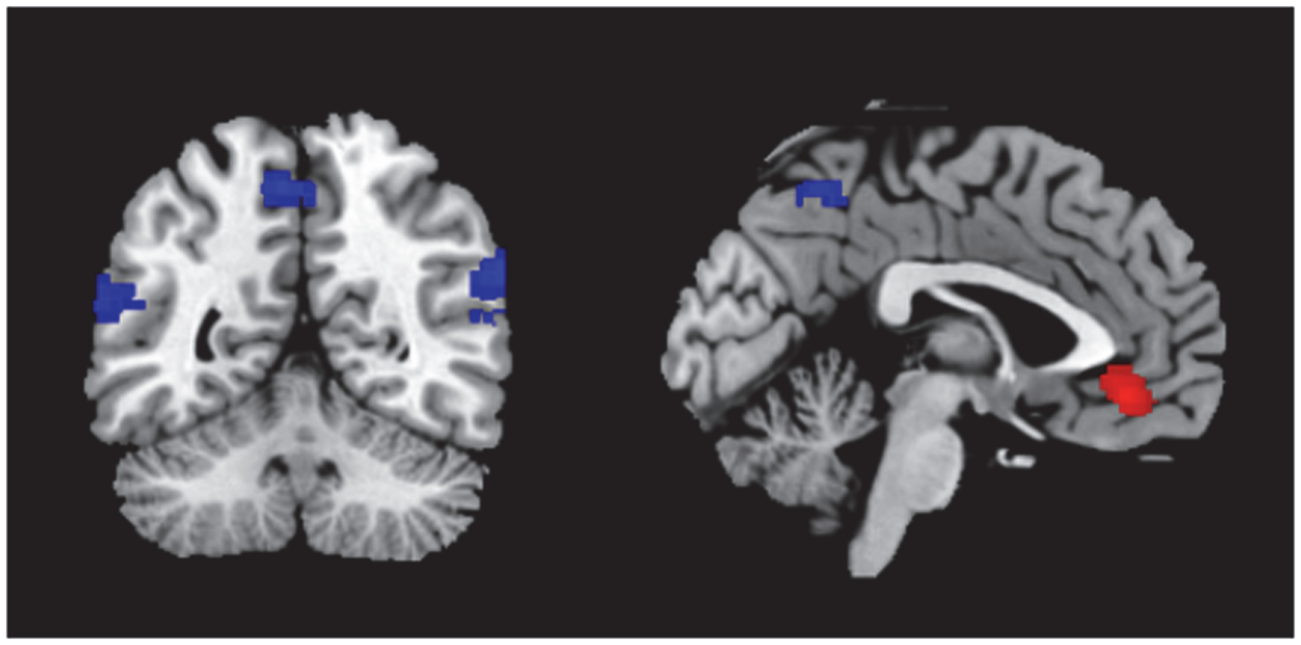
Brain maps illustrating activation clusters for the Mentalizing regressor contrasted against the Action regressor (red) and the Action regressor contrasted against the Mentalizing regressor (blue). All activations are corrected for the multiple comparisons at p<0.05.

Correlations between Mentalizing regressor activity in mentalizing regions and Action regressor activity in action regions. The r and p-values show that there are no significant correlations as observed in the ‘normal’ (non-reversed) data (reported in Table 2). Original p-values are reported (see Fig. 3 for an illustration). The responses in parts of the cortical motor network were summed over motor regions, as described in the methods section.

The mentalizing ROIs were co-activated as is evidenced by the correlations between mentalizing ROIs while participants listened to Mentalizing content (Table 4). The rTPJ and precuneus showed a significant correlation with each other during mentalizing content. The correlation of aMPFC with the other two regions of the mentalizing network was not statistically significant (Table 4). It is possible that the aMPFC stands out from the rest of the mentalizing regions (see Discussion), but we are cautious to draw this conclusion from the present findings since the non-significant correlations of aMPFC with rTPJ and precuneus are still sizeable (r = 0.39 and r = 0.42 respectively), which is in between medium and large effect size in Cohen’s convention [49]. These numbers cannot be taken as suggesting an absence of correlation.

**Table 4:** Correlations within sets of co-activated regions.

Mentalizing region 1	Mentalizing region 2	r	p-value
Right temporo-parietal junction	Precuneus	**0.745**	**<0.001**
	aMPFC	0.385	0.115
Precuneus	aMPFC	0.416	0.086

Action region 1	Action region 2	r	p-value
Left motor regions	Right motor regions	**0.606**	**0.008**

Correlations (as expressed by r- and p-value) between Mentalizing regressor activity in the different mentalizing regions (above), and between Action regressor activity in the different action regions (below). The r and p-values in bold typeface indicate significant correlations at the Bonferroni-corrected critical alpha value. The responses in parts of the cortical motor network were summed over regions, as described in the methods section.

The action ROIs (left and right motor regions) correlated significantly with each other while participants listened to Action descriptions (Table 4).

As a final control analysis, Mentalizing regressor activity in action ROIs was correlated with Action regressor activity in mentalizing ROIs (‘swapped correlations’), and no significant negative correlations were found, as was expected (all p>0.2). This is extra evidence that the negative correlations displayed in Fig. 2 and Table 2, are specific to the content of the stories driving mentalizing and action regions.

Activation levels in none of the ROIs were correlated with later memory for the stories, neither for the Mentalizing regressor, nor for the Action regressor (all p>0.12).

### Whole-brain analysis

**Fig 4:**
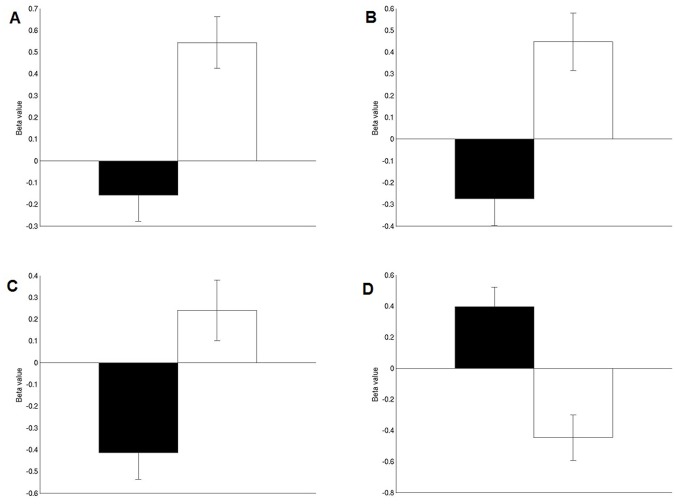
Bar plots showing mean activation levels (‘beta weights’) of each of the regressors (Mentalizing in black, Action in white) against baseline, in each of the regions found in the whole-brain analysis (Fig. 3). **A**) left superior temporal gyrus. **B**) right superior temporal gyrus. **C**) posterior cingulate cortex. **D**) anterior medial prefrontal cortex. Error bars represent standard error of the mean (s.e.m.).

Comparing activation for the Mentalizing regressor versus the Action regressor, a cluster in anterior medial prefrontal cortex was found, close to (and partially overlapping) the aMPFC region from the mentalizing localizer (Fig. 3; Table 5). For the Action regressor (versus the Mentalizing regressor), both left and right superior temporal gyrus were significantly activated, as well as the posterior cingulate cortex, extending into BA 7 (Fig. 3; Table 5). Activation levels of these four regions for both regressors compared to baseline are displayed in Fig. 4.

**Table 5:** Results of whole-brain analysis.

Contrast	x	y	z	Location / Brodmann Area	voxels	Max. T
Mentalizing>Action	-4	40	-10	Anterior Medial Prefrontal Cortex	286	4.61
Action>Mentalizing	-60	-58	22	L. superior temporal gyrus	553	4.27
	60	-60	26	R. superior temporal gyrus	330	4.79
	-6	-54	56	Posterior cingulate cortex	272	4.23

Displayed are the MNI coordinates of the peak voxel, a description of the localization of the activation, the number of activated voxels and the T-value of the maximally activated voxel in each cluster. See Fig. 3 for visualization of the results. All results are corrected for multiple comparisons at p<0.05.

## Discussion

Understanding fiction is more than an enjoyable pastime. Sharing narratives is a key component of human development (e.g. [2,3,50]), and there is evidence for the long-held conjecture that engagement with fiction renders people more empathic, presumably because of its enhancement of Theory-of-Mind abilities in the short and long term [1,51,52]. Another pointer towards the importance of fiction is that storytelling occurs in all cultures, and has existed throughout large part of human history [53]. Here we used fMRI to measure individual differences in narrative engagement on-line, measuring neural activation while people listened to excerpts from literary stories.

We show that participants employ mentalizing and motor simulation differently during fiction comprehension. There was a negative correlation between the activation in part of the cortical mentalizing network (anterior medial prefrontal cortex, aMPFC) for Mentalizing content in the story, and activation in the cortical motor network when participants listened to content related to Action. This suggests that there is a gradient among people in the way they engage with a narrative. Some rely mostly on mentalizing, others rely more on (sensori)-motor simulation, and yet others rely on both. This research gives more insight into individual differences in ways of engaging with fiction, by showing that there is no bimodal distribution with ‘simulators’ versus ‘non-simulators’, but that most readers rely on a specific type of simulation more than others. Some people are moved into a fiction story by mainly focusing on the thoughts and beliefs of others, whereas others pay more (implicit) attention to more concrete events such as action descriptions.

These findings add to recent insights about the two complementary systems for understanding actions and goals. A meta-analysis showed that the mirroring system (anterior intraparietal sulcus and premotor cortex) and the mentalizing system (TPJ, mPFC, and precuneus) play complementary roles, depending on how abstract the presented actions and/or goals are [19]. Another recent study, found that when participants were told to focus on the motive behind an action, the mentalizing system became active, whereas the mirroring system was activated when they focused on the implementation of an action [23].

Here, we observed this distinction between the two networks on the individual difference level, and within the broader scope of not just understanding actions and goals, but entire fictional stories. Apparently, when participants are explicitly told to use either kind of simulation, they will, but without instructions individual differences will play a larger role. Clearly, the fact that participants differed in their reliance on mentalizing or motor simulation during fiction comprehension does not mean that some are *incapable* of mentalizing or engaging in motor simulation. For instance, we did observe robust group-level activations to the Theory-of-Mind localizer task. The individual differences during narrative comprehension rather reflect an implicit preference, showing what participants do when processing fiction without additional task constraints.

It should be noted that the correlation we observed between Mentalizing and Action networks, only holds for one of the Mentalizing regions, namely the anterior medial prefrontal cortex. It is tempting to conclude that this region plays a privileged role during fiction comprehension, in comparison to the other parts of the mentalizing network (right TPJ and precuneus in the present study). Van Overwalle and Vandekerckhove [54] suggest that aMPFC and TPJ each fulfill specific roles during mentalizing. Anterior MPFC is thought to be activated by descriptions of stable characteristics and enduring traits, TPJ by more temporary intentions and beliefs [54]. Indeed, a large part of the mentalizing descriptions in our story materials consisted of personality trait descriptions. While it is possible that aMPFC shows up in our analysis for this reason, we are cautious with drawing strong conclusions on the respective roles of aMPFC and rTPJ / precuneus in the mentalizing network. The correlation values between right TPJ and precuneus and the motor cortex were still sizeable, and the correlations between the mentalizing regions (aMPFC, right TPJ, and precuneus) were all sizeable. Moreover, there is evidence from connectivity analyses that anterior MPFC and right TPJ are strongly connected [55,56]. Finally it is important to note that by characterizing the stimuli a priori (i.e., tagging the stories for action and mentalizing content) and by selecting relevant action and mentalizing ROIs based on functional localizers, the interpretation of the results does not rely strongly on reverse inference of the type that has been criticized before [57,58].

Our results nicely complement previous observations of individual differences in employment of mentalizing or sensori-motor simulation during story comprehension. Altmann and colleagues showed that individual differences in self-reported mentalizing correlate with the coupling of the aMPFC to other mentalizing regions during the reading of short stories labeled as fictional [27]. Other studies using behavioral measures show that individuals differ in how much they engage in sensori-motor simulation during language comprehension [29,30]. Our finding of individual differences is somewhat at odds with recent neuroimaging studies that found evidence for simulation across the study sample. For instance, Speer and colleagues found that premotor cortex was activated when participants heard action related descriptions, and Wallentin and colleagues showed involvement of visual motion areas when participants listened to descriptions of motion, and activations of the amygdala when participants listened to emotionally laden parts of a narrative [15,26,59]. In contrast, we do not observe consistent activations in motor regions with group-level statistics (the regions we find are, in fact, in line with [60]), but show that there are sizeable individual differences in participants’ sensitivity to action-related parts of the narrative. Note that we did observe activation of the aMPFC at the group level for listening to mentalizing-related parts of the story. One speculative reason for the difference across studies is in the difference in stimulus materials. Speer and colleagues [15] presented descriptions of activities of a 7 year old child (from ‘One boy’s day’, by Barker and Wright, [61]), and Wallentin and colleagues [26,59] used a classic fairy tale (‘The ugly duckling’, by H. C. Andersen) as materials. This is in contrast to the pieces of literary fiction, written for an adult audience, that we used here. The descriptions from ‘One boy’s day’ and ‘The ugly duckling’ could either be more concrete in their descriptions to start with, or participants may flexibly adapt to the genre they listen to (e.g. [62]), leading to a more heterogeneous response in the case of the different genres employed in the present study. The present data do not allow for testing these suggestions, and they should serve as inspiration for future research.

A final point is that our focus on sensori-motor simulation and mentalizing, two important factors in literary comprehension, is not meant to claim that there are no other ways to engage with fiction (e.g. [63]).

A remaining question is how the qualitative experience of fiction differs between people that rely more on the one kind of simulation compared to the other. It is likely that a qualitatively different type of engaging with a narrative leads to a qualitatively different experience of that narrative. We found no relation between memory performance and activation levels in any of the regions of interest, indicating that the difference in reading style that we observed does not lead to a difference in memory for the stories. The memory questions that we asked were however rather general, and perhaps not sensitive enough to pick up on fine-grained differences between participants. Moreover, it was not possible to qualify memory questions as being more indicative of mentalizing or motor simulation. This is in line with the findings by Happé and colleagues who observed different activations in medial prefrontal cortex in autistic as compared to healthy control participants, but did not observe differences in memory for stories [64]. Future research should investigate how individual differences in engaging with literature influence the phenomenological experience of a given narrative.

From a more methodological point of view, the present study adds to a recent line of work showing that using neuroimaging to gain more insight into discourse / narrative comprehension is feasible and can give important insights about language comprehension at the level of narrative (e.g. [15,26,65–72]).

In sum we provide on-line neural evidence for the existence of qualitatively different styles of engaging with fiction. Participants could be placed on a continuum of how much they relied on mentalizing or motor simulation while listening to literary fiction stories. People differ in how they are moved into a fiction world, and the current study qualifies part of how this is the case. Future work should be geared towards understanding what the consequences of these individual differences are for the understanding and appreciation of literature.
